# Prognostic Impact of AGR3 Protein Expression in Breast Cancer: A Systematic Review and Meta-analysis

**DOI:** 10.1055/s-0043-1772183

**Published:** 2023-11-09

**Authors:** Carolina Leão de Moraes, Carolina Rodrigues Mendonça, Natália Cruz e Melo, Fernanda Sardinha de Abreu Tacon, Jair Pereira de Melo Junior, Waldemar Naves do Amaral

**Affiliations:** 1 Faculdade de Medicina, Universidade Federal de Goiás, Goiânia, GO, Brazil; 2 Faculdade de Medicina, Universidade de Rio Verde, Rio Verde, GO, Brazil; 3 Escola Paulista de Medicina, Universidade Federal de São Paulo, São Paulo, SP, Brazil

**Keywords:** breast neoplasms, human AGR3 protein, immunohistochemistry, prognosis, survival, câncer de mama, proteína humana AGR3, imuno-histoquímica, prognóstico, sobrevida

## Abstract

**Objective** To investigate the clinicopathological significance and prognosis of the expression of the anterior gradient 3 (AGR3) protein in women with breast cancer.

**Data Sources** The PubMed, CINAHL, EMBASE, Scopus, and Web of Science databases were searched for studies published in English and without restrictions regarding the year of publication. The search terms were: *breast cancer* AND *anterior gradient 3* OR *AGR3 expression* .

**Study Selection** We included observational or interventional studies, studies on AGR3 protein expression by immunohistochemistry, and studies on invasive breast cancer. Case reports, studies with animals, and reviews were excluded. In total, 4 studies were included, containing 713 cases of breast cancer.

**Data Collection** Data were extracted on clinicopathological characteristics and survival. A meta-analysis of the prevalence of AGR3 expression was performed according to the clinicopathological characteristics, hazard ratios (HRs), and overall survival and disease-free survival.

**Data Synthesis** The expression of AGR3 was found in 62% of the cases, and it was associated with histological grade II, positivity of estrogen and progesterone receptors, low expression of ki67, recurrence or distant metastasis, and lumen subtypes. In patients with low and intermediate histological grades, AGR3 expression was associated with worse overall survival (HR: 2.39; 95% confidence interval [95%CI]: 0.628–4.159; *p* = 0.008) and worse disease-free survival (HR: 3.856; 95%CI: 1.026–6.686; *p* = 0.008).

**Conclusion** The AGR3 protein may be a biomarker for the early detection of breast cancer and predict prognosis in luminal subtypes. In addition, in patients with low and intermediate histological grades, AGR3 protein expression may indicate an unfavorable prognosis in relation to survival.

## Resumo

**Objetivo** Investigar o significado clinicopatológico e prognóstico da expressão da proteína *anterior gradient 3* (AGR3) em mulheres com câncer de mama.

**Fontes de Dados** Utilizamos as bases de dados PubMed, CINAHL, EMBASE, Scopus e Web of Science para pesquisar estudos em inglês, sem restrições quanto ao ano de publicação. Os termos buscados foram: *breast cancer* AND *anterior gradient 3* OR *AGR3 expression* .

**Seleção dos Estudos** Foram incluídos estudos observacionais ou intervencionais, estudos sobre a expressão da proteína AGR3 por imuno-histoquímica, e estudos sobre câncer de mama invasivo. Excluíram-se relatos de casos, estudos com animais e revisões. Quatro estudos foram selecionados, que continham 713 casos de câncer de mama.

**Coleta de Dados** Foram extraídos dados relativos a características clinicopatológicas e sobrevida. A metanálise da prevalência da expressão de AGR3 foi realizada conforme as características clinicopatológicas, razões de risco (RRs) e sobrevida global (SG) e sobrevida livre de doença (SLD).

**Síntese dos Dados** Encontrou-se expressão de AGR3 em 62% dos casos, que se associou com grau histológico II, positividade de receptores de estrogênio e progesterona, baixa expressão de ki67, recorrência ou metástase à distância e subtipos luminais. Em pacientes com graus histológicos baixo e intermediário, a expressão de AGR3 conferiu pior SG (RR: 2,39; intervalo de confiança de 95% [IC95%]: 0,628–4,159; *p* = 0,008) e pior SLD (RR: 3,856; IC95%: 1,026–6,686; *p* = 0,008).

**Conclusão** A AGR3 pode ser um biomarcador para a detecção precoce do câncer de mama e predizer o prognóstico em subtipos luminais. Em graus histológicos baixo e intermediário, a expressão da proteína AGR3 pode indicar um prognóstico desfavorável em relação à sobrevida.

## Introduction

Breast cancer is of great epidemiological relevance due to the high rates of mortality and morbidity in the world. In 2020, breast cancer represented the main cause of death due to cancer among women, affecting ∼ 2.3 million new cases. 1

With advances in large-scale techniques, gene expression signatures capable of stratifying breast cancer into molecular subtypes that aid in diagnosis, response to treatment, and prognosis have been proposed. Through these analyses, breast cancers have been stratified into four subtypes: luminal A, luminal B, human epidermal growth factor receptor-2 positive (HER2 + ), and basal-like. 2 3 Despite the translational application of the molecular stratification of breast cancer, many patients develop resistance to treatments and recurrence, which instigates research that seeks new biomarkers of prognosis and response to chemotherapy. 4

Anterior gradient 3 (AGR3) is a member of the protein disulfide isomerase (PDI) gene family. In recent years, the protein encoded by this gene has attracted the attention of researchers due to its role in the process of carcinogenesis. 5 The clinical relevance of AGR3 has been demonstrated in several cancers, including ovarian cancer, 6 7 prostate cancer, 8 intrahepatic cholangiocarcinoma, hepatocellular carcinoma, 9 and breast cancer. 5 10 11 12 13 Scientific evidence has suggested that AGR3 has prognostic value in ovarian and breast cancers. 6 14 In ovarian cancer, AGR3 is upregulated in the serous 6 and clear-cell subtypes, 7 and high levels of AGR3 are a predictor of better survival. 6 In breast cancer, AGR3 is considered a potential biomarker for early detection in blood and tissue 5 11 and for prognosis. 13

The role of AGR3 in the clinic of breast cancer remains nuclear, 5 12 due to the limited studies that present the clinicopathological and prognostic relevance of this protein. 5 However, the evidence suggest that AGR3 may be associated with oncogenesis, and it has been pointed out as a potential therapeutic target and prognostic biomarker for patients with breast cancer. According to these precedents, the objective of the present systematic review and meta-analysis was to investigate the clinicopathological significance and prognosis of the expression of the AGR3 protein in women with breast cancer.

## Materials and Methods

The present systematic review and meta-analysis was performed following the guidelines of the Preferred Reporting Items for Systematic Reviews and Meta-Analysis (PRISMA) 15 statement and the Meta-analysis of Observational Studies in Epidemiology (MOOSE) group. 16 In adittion, the study was registered in the International Prospective Register of Systematic Reviews (PROSPERO) database (CRD42021244277).

An electronic search was performed on the PubMed, CINAHL, EMBASE, Scopus, and Web of Science databases. Searches on Google Scholar and the primary study reference list were also conducted to identify additional studies. The search terms were: *breast cancer* AND *anterior gradient 3* OR *AGR3 expression* . The search strategies for each database are presented in chart 1 .

**Chart 1:** Search strategies used in each database

Databases	Search Strategy
MEDLINE/PubMed	*Search: (“breast neoplasms”[MeSH Terms] OR (“breast”[All Fields] AND “neoplasms”[All Fields]) OR “breast neoplasms”[All Fields] OR (“breast”[All Fields] AND “cancer”[All Fields]) OR “breast cancer”[All Fields]) AND (((“anterior”[All Fields] OR “anteriores”[All Fields] OR “anteriorization”[All Fields] OR “anteriorized”[All Fields] OR “anteriors”[All Fields]) AND (“gradient”[All Fields] OR “gradient s”[All Fields] OR “gradients”[All Fields]) AND “3”[All Fields]) OR (“AGR3”[All Fields] AND (“express”[All Fields] OR “expresse”[All Fields] OR “expresses”[All Fields] OR “expressing”[All Fields] OR “expressions”[All Fields] OR “gene expression”[MeSH Terms] OR (“gene”[All Fields] AND “expression”[All Fields]) OR “gene expression”[All Fields] OR “expressed”[All Fields] OR “expression”[All Fields])))* ***Total: 32***
CINAHL	*Boolean/Phrase: Breast Cancer AND AGR3 OR Anterior gradient 3* ***Total: 3***
EMBASE	*1 breast cancer/394079* *2 Anterior gradient 3.mp./12* *3 AGR3.mp./94* *4 2 or 3/94* *5 1 and 4/12* ***Total: 12***
Scopus	*TITLE-ABS-KEY ((breast cancer) AND (Anterior gradient 3 OR AGR3))* ***Total: 29***
Web of Science	*((breast cancer) AND (AGR3 OR Anterior gradient 3))* ***Total: 40***

The inclusion criteria were: 1) observational or interventional studies involving the expression of the AGR3 protein in women with breast cancer; 2) studies evaluating the prognostic capacity of the AGR3 protein expression by immunohistochemistry; and 3) studies on invasive breast cancer; moreover, there were no restrictions regarding language or the year of publication of the studies. The following were excluded: dissertations, theses, case reports, studies with animals, reviews, editorials, letters to the editor, and duplicate studies found in more than one database.

Titles and abstracts were read using the Rayyan (Rayyan Systems Inc., Cambridge, MA, United States) software. The studies retrieved were analyzed by the authors, the selected articles were read in full, and the inclusion and exclusion criteria were applied. Doubts and/or disagreements about the articles were discussed by the research team.

The following data were extracted: author and year of publication, study design, country, number of patients, age of the patients, methods of evaluation and results of AGR3 expression in women with breast cancer, and clinical results (clinicopathological characteristics and survival).The clinicopathological characteristics included: age, histological grade, estrogen receptor (ER), progesterone receptor (PR), HER2, K _i_ −67, recurrence or distant metastasis, and molecular subtypes (luminal A, luminal B, HER2 + , and triple-negative). The data collected on survival were: overall survival (OS) and disease-free survival (DFS).

The risk of bias was assessed using the Cochrane's Risk of Bias in Non-Randomized Studies – of Interventions (ROBINS-I) tool. 17 Eight methodological domains were evaluated: 1) bias due to confounding; 2) bias in the selection of participants into the study; 3) bias in the measurement of interventions; 4) bias due to departures from the intended interventions; 5) bias due to missing data; 6) bias in the measurement of outcomes; 7) bias in the selection of the reported result; and 8) overall bias. Each domain was classified as presenting “low risk of bias,” “moderate risk of bias,” “serious risk of bias,” and “critical risk of bias.”

The methodological quality of the studies was evaluated using the software application of the Grading of Recommendations, Assessment, Development, and Evaluations (GRADE) approach ( https://gdt.gradepro.org/app/#), ^18,19^ which considers four categories: high, moderate, low, and very low quality. 20 Thus, the quality of the evidence was classified into these aforementioned categories.

Meta-analyses were conducted using the random effects model on coded data stratified by the expression of AGR3. The meta-analysis of prevalence of AGR3 expression was performed according to clinicopathological characteristics, hazard ratios (HRs) and OS and DFS analyses. The data were expressed graphically in forest plots, with estimates on the prevalence and HRs with 95% confidence intervals (95%CIs). The degree of heterogeneity among the studies was estimated by the statistical values of I ^2^ : < 25% – low heterogeneity;, 25% to 50% – moderate heterogeneity; and > 50% –high heterogeneity. 21 Publication bias was assessed using the Egger test and funnel plot asymmetry. All analyses were performed using the STATA (StataCorp LLC, College Station, TX, United States) software, version 16.0.

## Results

**Fig. 1:**
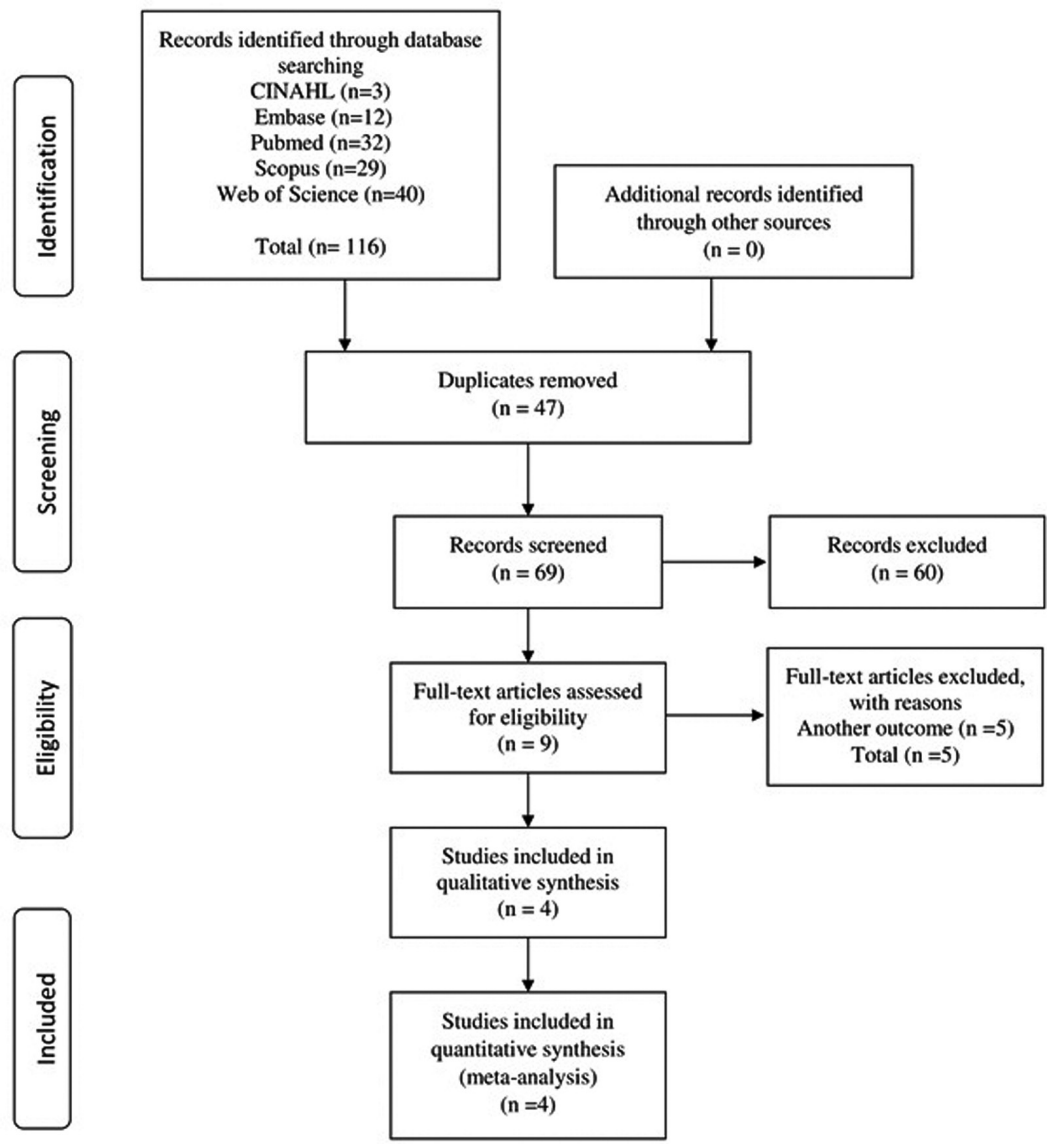
Flowchart of the process of selection of studies.

A total of 116 articles were identified in the 5 databases evaluated. After the careful process of screening and removing duplicates, 69 articles were selected and had their titles and abstracts read; then, 9 articles were selected for full-text reading, and 4 articles presented the eligibility criteria and were included in the present systematic review and meta-analysis. 5 10 11 13 The article selection process is illustrated in a flowchart prepared in accordance with the PRISMA statement ( Fig. 1 ).

The excluded articles and the reasons for the exclusions are presented in chart 2 .

**Chart 2:** Excluded articles and reason for exclusion

Number	Title	Reason for exclusion
1	Jian, Lei et al. AGR3 promotes estrogen receptor-positive breast cancer cell proliferation in an estrogen-dependent manner. *Oncology Letters* , v. 20, n. 2, p. 1,441–1,451, 2020.	Another outcome
2	Umesh, Anita et al. Identification of AGR3 as a potential biomarker though public genomic data analysis of triple-negative (TN) versus triple-positive (TP) breast cancer (BC). *Journal of Clinical Oncology* , v. 30, n. 27, supplement 31, 2012.	Another outcome
3	Obacz, Joanna et al. The role of AGR2 and AGR3 in cancer: similar but not identical. *European Journal of Cell Biology* , v. 94, n. 3–4, p. 139–147, 2015.	Another outcome
4	Obacz, Joanna et al. Extracellular AGR3 regulates breast cancer cells migration via Src signaling. *Oncology Letters* , v. 18, n. 5, p. 4,449–4,456, 2019.	Another outcome
5	PERSSON, Staffan et al. Diversity of the protein disulfide isomerase family: identification of breast tumor induced Hag2 and Hag3 as novel members of the protein family. *Molecular Phylogenetics and Evolution* , v. 36, n. 3, p. 734–740, 2005.	Another outcome

**Fig. 2:**
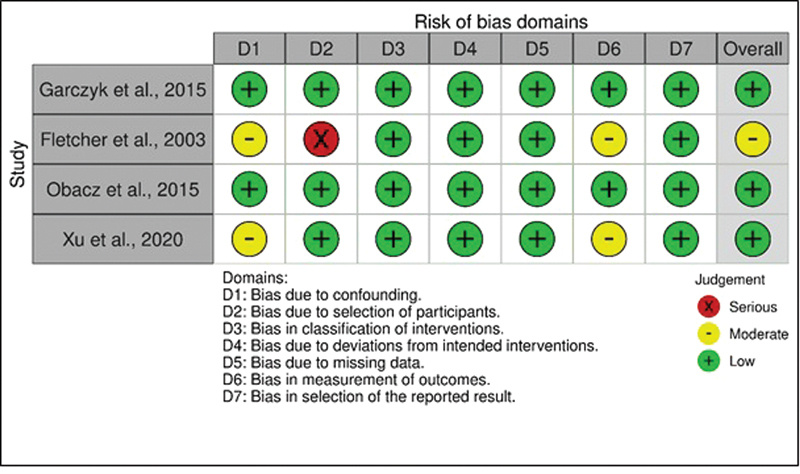
Summary of the authors' judgments about each item of the risk of bias assessment for each included study.

Four studies were evaluated using the GRADE approach and ROBINS-I. The GRADE score indicated that three studies showed moderate quality of evidence 5 11 13 and one study, showed poor quality ( Table 1 ). 10 The results of the risk of bias assessment are shown in Fig. 2 .

**Table 1:** Characteristics of the studies included in the systematic review and meta-analysis

Reference	N/age	Study design/ follow-up	Immunohistochemical staining	AGR3 expression	Conflict of interests	Ethical approval	Quality of the evidence (GRADE)
Obacz et al., 2015 5 (Czech Republic)	129/29 to 84 (median: 57) years	Cohort/ 10 years	Tumor samples were fixed in 10% neutral buffered formalin for 24 hours and then embedded in paraffin wax. Immunohistochemical analysis was performed on 4-μm thick sections cut from formalin-fixed paraffin-embedded archival tissue blocks, mounted on slides, deparaffinized in xylene, and rehydrated in phosphate-buffered saline through a graded ethanol series.	104/129	No	Yes	⊕⊕⊕⊝moderate
Fletcher et al., 2003 10 (United Kingdom)	58/< 50 years versus 50 years	Not reported	Immunohistochemical analysis was performed on formalin-fixed paraffin-embedded tissue microarrays containing 1-mm sections of breast carcinoma tissues from 60 donors. Slides were deparafinized by two 5-minute washes in xylene, then rehydrated through successive graded ethanol solutions and washed for 5 minutes in phosphate-buffered saline.	43/58	No	Yes	⊕⊕⊝⊝ Low
Garczyk et al., 2015 11 (Germany)	190 **Age:** Less than 54,5 years of age versus 54,5 years	Cohort 138.5 (range: 1–218) months	Immunohistochemical analysis was performed according to the manufacturer's instructions (DAKO 5001; DAKO, Glostrup, Denmark). Heat-induced epitope retrieval was performed in 10-mM citrate buffer (pH 6.0) for 10 minutes using a pressure cooker. Formalin-fixed, paraffin-embedded sections (3 μm) were incubated for 45 minutes with mouse monoclonal anti-AGR3-antibody (1:1000; ab82400, Abcam, Cambridge, United Kingdom).	104/190	No	Yes	⊕⊕⊕⊝moderate
Xu et al., 2020 13 (China)	336*/52 years, which ranged from 28 to 89 years	Cohort/ 5–146 months	All procedures were performed in Benchmark XT (Roche, Basel, Switzerland). Antigen retrieval was performed in citrate buffer at 121°C for 2 minutes 15 seconds. After retrieval, sections were steeped in 3% H2O2 buffer for 25 minutes and in 10% goat serum buffer for 25 minutes. Then, the sections were steeped in primary antibody against AGR3 (1:100, HPA053942, Sigma, St. Louis, MO, United States) at 4°C for 1 night.	129/336	No	Yes	⊕⊕⊕⊝moderate

Abbreviations: AGR3, anterior gradient 3; GRADE, Grading of Recommendations, Assessment, Development, and Evaluations.

Note: *Invasive ductal carcinoma. GRADE Working Group grades of evidence: high quality – further research is very unlikely to change our confidence in the estimate of effect; moderate quality – further research is likely to have an important impact on our confidence in the estimate of effect and may change the estimate; low quality – further research is very likely to have an important impact on our confidence in the estimate of effect and is likely to change the estimate; and very low quality – we are very uncertain about the estimate.

**Fig. 3:**
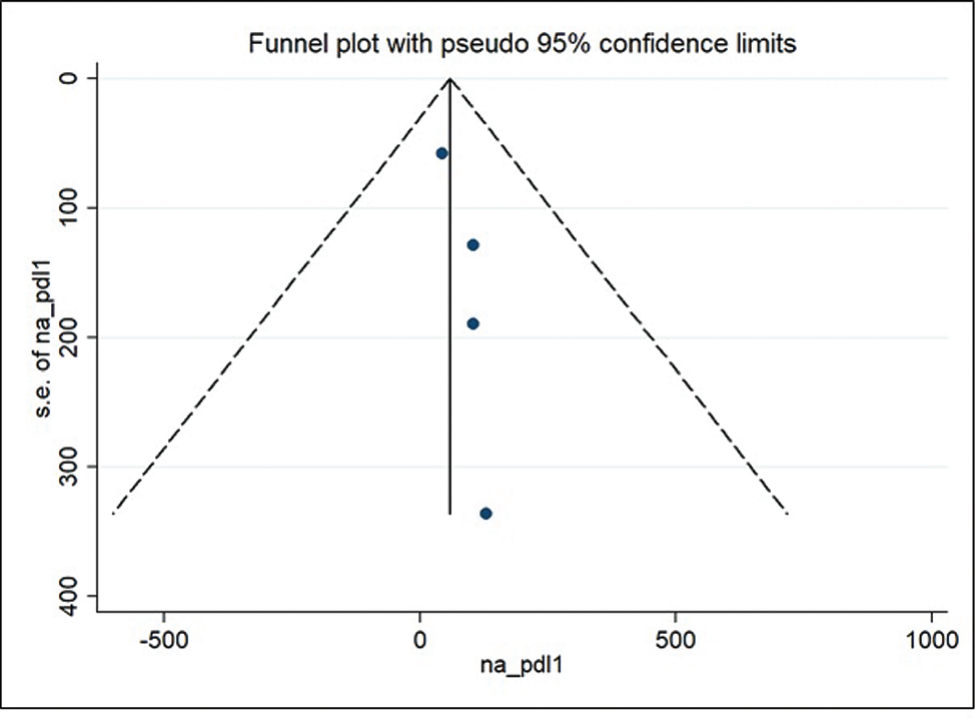
Funnel plot for the studies included in the meta-analysis.

A funnel plot was developed to assess the publication bias ( Fig. 3 ). This analysis revealed a symmetrical pattern, and there was no evidence of a notable publication bias that could confuse the results. The Egger test ruled out the apparent bias in studies that analyzed the expression of AGR3 in women with breast cancer ( *p* = 0.105).

The present systematic review included four studies, 5 10 11 13 comprising a total of 713 cases of breast cancer from Germany, 11 the United Kingdom, 10 the Czech Republic, 5 and China. 13 The characteristics of each study are shown in table 1 .

**Fig. 4:**
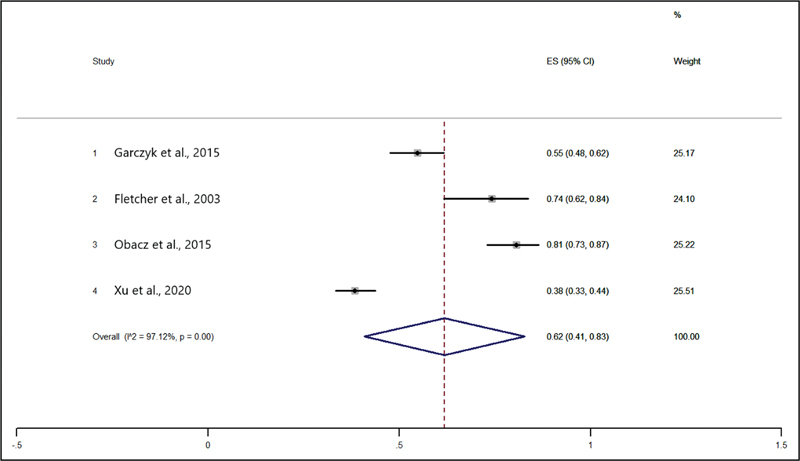
Forest plots of the prevalence (%) of anterior gradient 3 (AGR3) expression in women with breast cancer.

The studies included showed results of the prevalence of AGR3 expression in women with breast cancer that were included in the meta-analysis. 5 10 11 13 The prevalence of AGR3 expression was of 62%, as shown in Fig. 4 .

The results of the meta-analysis of the prevalence of AGR3 expression according to the clinicopathological variables are summarized in Table 2 . The type-II histological grade (65%; *p* = 0.048; I ^2^ = 95.33%), 5 11 13 ER positivity (72%; *p* = 0.000; I ^2^ = 98.53%), 5 11 13 PR positivity (69%; *p* = 0.000; I ^2^ = 96.74%), 5 11 13 negativity of Ki-67 expression (52%; *p* = 0.015; I ^2^ = 90.16%), 5 13 recurrence or distant metastasis (55%; *p* = 0.001; I ^2^ = 0%). 13 and luminal subtypes A (46%) and B (48%) ( *p* = 0.000; I ^2^ = 94.06%) 13 have been associated with positive AGR3 expression in women with breast cancer.

**Table 2:** Meta-analysis of AGR3 expression and clinicopathological features of breast cancer

Analysis	Proportion (%) Of AGR3	*p-value* for overall effect	Heterogeneity I ^2^ (%)/ *p* -value	References
Age (in years)				
< 50	0.44%	0.068	0%/ *p* = 0.00	13
≥ 50	0.34%			
Histological grade				
I	0.44%	0.048	95.33%/ *p* = 0.00	5 11 13
II	0.65%			
III	0.24%			
Estrogen receptor				
(–)	0.12%	0.000	98.53%/ *p* = 0.00	5 11 13
(+)	0.72%			
Progesterone receptor				
(–)	0.22%	0.000	96.74%/ *p* = 0.00	5 11 13
(+)	0.69%			
MIB1/Ki-67 expression				
Low	0.52%	0.015	90.16%/ *p* = 0.00	5 13
High	0.41%			
HER2				
(–)	0.60%	0.059	92.36%/ *p* = 0.00	5 11 13
(+)	0.33%			
*Recurrence or distant metastasis*				
No	0.34%	0.001	.%, *p* = 0.00	13
Yes	0.55%			
Molecular subtypes				
Luminal A	0.46%	0.000	94.06%, *p* = 0.00	13
Luminal B	0.48%			
HER2+	0.11%			
Triple-negative	0.15%			

Abbreviations: AGR3, anterior gradient 3; HER2, human epidermal growth factor receptor-2.

Notes: I ^2^ : heterogeneity between groups; *p* < 0.05: statistically significant; observation: prevalence data and I ^2^ were extracted from the meta-analysis graphs.

Information about the association regarding AGR3 expression and OS 5 11 13 and DFS 5 13 in women with breast cancer are shown in Table 3 .

**Table 3:** Association between survival and AGR3 expression in women with breast cancer

*Global survival*	
Obacz et al., 2015 5	Determination of overall survival by Kaplan–Meier analysis in patients with “high” AGR3 expression and patients with “low” AGR3 expression using Breslow test ( *p* = 0.111).
Garczyk et al., 2015 11	Patients with low and intermediate grade tumors showing high AGR3 expression had an unfavorable outcome (mean tumor-specific survival: 142.5 ± 9.6 months; 95%CI: 123.8–161.2) compared with those with low AGR3 expression (mean tumor-specific survival: 181.7 ± 10.1 months; 95%CI: 162.0–201.4). The Cox regression model confirmed AGR3 to be a putative independent marker of unfavorable prognosis in low- and intermediate-grade breast tumors (multivariate HR: 2.186; 95%CI: 1.008–4.740; *p* < 0.05).
Xu et al., 2020 13	IDC patients of grades I-II: in the multivariate Cox regression analysis, we found that AGR3 expression was an independent predictor for overall survival (HR: 4.161; 95%CI: 1.406–12.312; *p* < 0.010).
***Disease-free survival***	
Obacz et al., 2015 5	Determination of progression-free survival by Kaplan–Meier analysis in patients with “high” AGR3 expression (more than 50% of positive cells) and patients with “low” AGR3 expression (less than 50% of positive cells) using Breslow test ( *p* = 0.037)
Xu et al., 2020 13	IDC patients of grades I-II: in the multivariate Cox regression analysis, we found that AGR3 expression was an independent predictor for disease-free survival (HR: 3.856; 95%CI: 1.953–7.613; *p* < 0.001)

Abbreviations: 95%CI, 95% confidence interval; AGR3, anterior gradient 3; HR, hazard ratio; IDC, invasive ductal carcinoma.

**Fig. 5:**
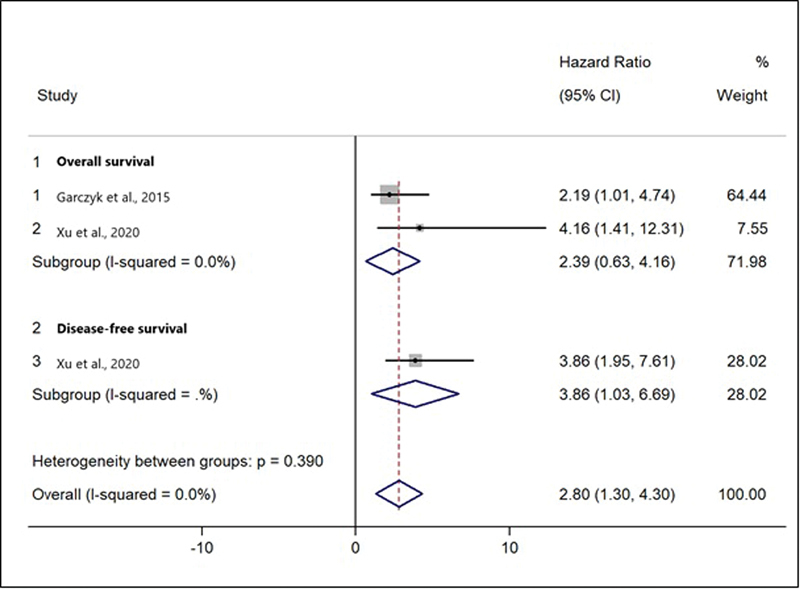
Forest plots of the hazard ratios (HRs) for survival based on the expression of AGR3.

Fig. 5 shows the combined HR and forest plots for survival based on AGR3 expression. The result of the meta-analysis revealed that AGR3 expression was associated with a worse OS (HR: 2.39; 95%CI = 0.63–4.16; *p* = 0.008) 11 13 and DFS (HR: 3.86; 95%CI = 1.03–6.69; *p* = 0.008). 13 In addition, the final result of the meta-analysis indicated that AGR3 expression was associated with poorer survival in low- and intermediate-grade tumors (HR: 2.80; 95%CI = 1.30–4.30; *p* = 0.000) ( Fig. 5 ). No heterogeneity was observed among the included studies (I ^2^ = 0.0%).

## Discussion

Currently, there are studies that point out the potential of AGR3 in breast carcinogenesis. 5 10 11 13 The present systematic review and meta-analysis evaluated 713 cases and found a high prevalence of AGR3 protein expression in patients with breast cancer. The AGR3 protein was associated with positivity of estrogen and progesterone receptors, histological grade II, low expression of Ki-67, recurrence or distant metastasis, and luminal subtypes. In addition, AGR3 has a prognostic value for conferring worse OS and DFS in patients with histological grades I and II.

In the present study, we observed a prevalence of AGR3 positivity of 62% in breast cancer, which demonstrates the relevance of this protein in breast carcinogenesis, confirming the predominant expression previously reported. 5 11 The expression of AGR3 in general seems to be associated with a less aggressive phenotype, with hormone receptor positivity, low histological grade, and low proliferation rate. The association of AGR3 with the positivity of estrogen and progesterone receptors demonstrates that there is a close relationship between AGR3 and the luminal subtypes. 5 11 13 This interaction has often been reported 10 11 13 14 and, in conjunction with the other histological and proliferative characteristics, it suggests that AGR3 is associated with less aggressive cancers that are generally responsive to treatment and therefore have a favorable result. 5

The findings of the present study indicate that the expression of AGR3 was reduced for the triple-negative subtype of breast cancer. This finding is in line with that of another recent study that analyzed AGR3 mRNA gene expression in breast cancer cell tissues. 12

Although AGR3 expression has been associated with less aggressive clinicopathological features in breast cancer, paradoxically, in the present study, we have identified the association of AGR3 expression with distant recurrence and/or metastasis and an unfavorable outcome in relation to survival, demonstrating the complexity of that molecule. In luminal subtype B, high AGR3 expression was associated with high risk of recurrence and metastasis and poor prognosis in patients with invasive breast carcinoma. 13 In addition, AGR3 appears to have protumor functions in breast cancer, by regulating the adhesion and migration processes of tumor cells through the activation of Src kinases. 22

The result of the meta-analysis revealed that AGR3 expression was associated with worse OS 11 13 and DFS 13 in low- and intermediate-grade cancers. Garczyk et al. 11 suggested a prooncogenic impact of AGR3 in tumors of low and intermediate histological grade, and they also highlighted the potential of AGR3 for the early detection of breast cancer, with high specificity (of 92.5%) and sensitivity (of 35%).

Regarding the therapeutic potential, a recent study 13 concluded that, in patients with luminal subtype B and histological grades I and II, the AGR3 expression conferred an unfavorable prognosis and suggested that this patients should be treated with 5-fluoropyrimidine chemotherapy, but not taxane. The authors 13 warned that AGR3 can promote tumor progression, through processes of proliferation, invasion, and resistance to chemotherapy.

The present research confirmed the association of AGR3 with important features in the breast cancer clinic, such as hormone receptors, proliferation index, and prognosis. The AGR3 protein may be a biomarker of poor prognosis in low- and intermediate-grade tumors and luminal subtypes, representing an interesting tool for the clinical management of this population. Considering the recent development in cancer research, understanding the functions of AGR3 would be inevitable for the development of predictive tools for prognosis and new target therapies. 14 In addition, AGR3 can serve as a biomarker in the early detection of breast cancer and to predict the clinical outcome. 14

The present systematic review and meta-analysis has certain limitations. Firstly, we included only four studies, and the size of the sample of one of them was small. Secondly, although no evidence of publication bias was verified by the Egger test, exclusion of unpublished data and gray literature may have introduced selection bias in the analysis.

The present review also has strengths. Firstly, the research was conducted in five important databases in health sciences, and there was scientific rigor in the analysis process. Secondly, there was no restrictions on the year of publication and language. Thirdly, all references included were full-text articles published in peer-reviewed journals. In addition, the studies reported no conflicts of interest and were approved by ethics committees. Although the results of the present systematic review should be interpreted with caution, the evidence presented at the moment may serve as a guide for future research and also for the clinical practice.

## Conclusion

The AGR3 protein may be a biomarker to predict prognosis in luminal subtypes. In addition, in patients with tumors of low and intermediate histological grades, AGR3 expression may indicate unfavorable prognosis in relation to OS and DFS. Although the present study indicates that AGR3 may be promising to predict prognosis in luminal subtypes, we highlight the need for more high-quality studies to confirm these findings, and these should be considered when making decisions regarding the prediction of diagnosis and prognosis in breast cancer.

## References

[1] Sung H Ferlay J Siegel R L Laversanne M Soerjomataram I Jemal A Bray F Global cancer statistics 2020: GLOBOCAN estimates of incidence and mortality worldwide for 36 cancers in 185 countriesCancer J Clin2021 10.3322/caac.21660 33538338

[2] Perou C M Sørlie T Eisen M B van de Rijn M Jeffrey S S Rees C A Molecular portraits of human breast tumours Nature 2000 406 6797 747 752 10963602 10.1038/35021093

[3] Sørlie T Tibshirani R Parker J Hastie T Marron J S Nobel A Repeated observation of breast tumor subtypes in independent gene expression data sets Proc Natl Acad Sci U S A 2003 100 14 8418 8423 12829800 10.1073/pnas.0932692100 PMC166244

[4] Riggio A I Varley K E Welm A L The lingering mysteries of metastatic recurrence in breast cancer Br J Cancer 2021 124 01 13 26 33239679 10.1038/s41416-020-01161-4 PMC7782773

[5] Obacz J Brychtova V Podhorec J Fabian P Dobes P Vojtesek B Hrstka R Anterior gradient protein 3 is associated with less aggressive tumors and better outcome of breast cancer patients OncoTargets Ther 2015 8 1523 1532 10.2147/OTT.S82235 PMC4485854 26170690

[6] King E R Tung C S Tsang Y TM Zu Z Lok T MG Deavers M T The anterior gradient homolog 3 (AGR3) gene is associated with differentiation and survival in ovarian cancer Am J Surg Pathol 2011 35 06 904 912 21451362 10.1097/PAS.0b013e318212ae22 PMC3095702

[7] Samanta S Tamura S Dubeau L Mhawech-Fauceglia P Miyagi Y Kato H Expression of protein disulfide isomerase family members correlates with tumor progression and patient survival in ovarian cancer Oncotarget 2017 8 61 103543 103556 29262583 10.18632/oncotarget.21569 PMC5732749

[8] Bu H Schweiger M R Manke T Wunderlich A Timmermann B Kerick M Anterior gradient 2 and 3–two prototype androgen-responsive genes transcriptionally upregulated by androgens and by oestrogens in prostate cancer cells FEBS J 2013 280 05 1249 1266 23294566 10.1111/febs.12118

[9] Brychtova V Zampachova V Hrstka R Fabian P Novak J Hermanova M Vojtesek B Differential expression of anterior gradient protein 3 in intrahepatic cholangiocarcinoma and hepatocellular carcinoma Exp Mol Pathol 2014 96 03 375 381 24747240 10.1016/j.yexmp.2014.04.002

[10] Fletcher G C Patel S Tyson K Adam P J Schenker M Loader J A hAG-2 and hAG-3, human homologues of genes involved in differentiation, are associated with oestrogen receptor-positive breast tumours and interact with metastasis gene C4.4a and dystroglycan Br J Cancer 2003 88 04 579 585 12592373 10.1038/sj.bjc.6600740 PMC2377166

[11] Garczyk S von Stillfried S Antonopoulos W Hartmann A Schrauder M G Fasching P A AGR3 in breast cancer: prognostic impact and suitable serum-based biomarker for early cancer detection PLoS One 2015 10 04 e0122106 25875093 10.1371/journal.pone.0122106 PMC4398490

[12] Jian L Xie J Guo S Yu H Chen R Tao K Yang C AGR3 promotes estrogen receptor-positive breast cancer cell proliferation in an estrogen-dependent manner Oncol Lett 2020 20 02 1441 1451 32724387 10.3892/ol.2020.11683 PMC7377037

[13] Xu Q Shao Y Zhang J Zhang H Zhao Y Liu X Anterior Gradient 3 Promotes Breast Cancer Development and Chemotherapy Response Cancer Res Treat 2020 52 01 218 245 31291711 10.4143/crt.2019.217 PMC6962492

[14] Obacz J Takacova M Brychtova V Dobes P Pastorekova S Vojtesek B Hrstka R The role of AGR2 and AGR3 in cancer: similar but not identical Eur J Cell Biol 2015 94(3-4):139 147 25666661 10.1016/j.ejcb.2015.01.002

[15] PRISMA Group Moher D Liberati A Tetzlaff J Altman D G Preferred reporting items for systematic reviews and meta-analyses: the PRISMA statement PLoS Med 2009 6 07 e1000097 19621072 10.1371/journal.pmed.1000097 PMC2707599

[16] Stroup D F Berlin J A Morton S C Olkin I Williamson G D Rennie D Meta-analysis of Observational Studies in EpidemiologyA Proposal for Reporting JAMA 2000 283 2008 2012 10789670 10.1001/jama.283.15.2008

[17] Sterne J AC Hernán M A Reeves B C Savović J Berkman N D Viswanathan M ROBINS-I: a tool for assessing risk of bias in non-randomised studies of interventions BMJ 2016 355 i4919 27733354 10.1136/bmj.i4919 PMC5062054

[18] Carrasco-Labra A Brignardello-Petersen R Santesso N Neumann I Mustafa R A Mbuagbaw L Improving GRADE evidence tables part 1: a randomized trial shows improved understanding of content in summary of findings tables with a new format J Clin Epidemiol 2016 74 7 18 26791430 10.1016/j.jclinepi.2015.12.007

[19] Guyatt G Oxman A D Akl E A Kunz R Vist G Brozek J GRADE guidelines: 1. Introduction-GRADE evidence profiles and summary of findings tables J Clin Epidemiol 2011 64 04 383 394 21195583 10.1016/j.jclinepi.2010.04.026

[20] Balshem H Helfand M Schünemann H J Oxman A D Kunz R Brozek J GRADE guidelines: 3. Rating the quality of evidence J Clin Epidemiol 2011 64 04 401 406 21208779 10.1016/j.jclinepi.2010.07.015

[21] Higgins J PT Thompson S G Deeks J J Altman D G Measuring inconsistency in meta-analyses BMJ 2003 327 7414 557 560 12958120 10.1136/bmj.327.7414.557 PMC192859

[22] Obacz J Sommerova L Sicari D Durech M Avril T Iuliano F Extracellular AGR3 regulates breast cancer cells migration via Src signaling Oncol Lett 2019 18 05 4449 4456 31611954 10.3892/ol.2019.10849 PMC6781763

